# Prediction of Breast Cancer Histological Outcome by Radiomics and Artificial Intelligence Analysis in Contrast-Enhanced Mammography

**DOI:** 10.3390/cancers14092132

**Published:** 2022-04-25

**Authors:** Antonella Petrillo, Roberta Fusco, Elio Di Bernardo, Teresa Petrosino, Maria Luisa Barretta, Annamaria Porto, Vincenza Granata, Maurizio Di Bonito, Annarita Fanizzi, Raffaella Massafra, Nicole Petruzzellis, Francesca Arezzo, Luca Boldrini, Daniele La Forgia

**Affiliations:** 1 Radiology Division, Istituto Nazionale Tumori—IRCCS—Fondazione G. Pascale, 80131 Naples, Italy; t.petrosino@istitutotumori.na.it (T.P.); m.barretta@istitutotumori.na.it (M.L.B.); a.porto@istitutotumori.na.it (A.P.); v.granata@istitutotumori.na.it (V.G.); 2 Medical Oncology Division, Igea SpA, 80013 Naples, Italy; r.fusco@igeamedical.com (R.F.); e.dibernardo@igeamedical.com (E.D.B.); 3 Pathology Division, Istituto Nazionale Tumori—IRCCS—Fondazione G. Pascale, 80131 Naples, Italy; m.dibonito@istitutotumori.na.it; 4 Direzione Scientifica—IRCCS Istituto Tumori Giovanni Paolo II, Via Orazio Flacco 65, 70124 Bari, Italy; a.fanizzi@oncologico.bari.it; 5 SSD Fisica Sanitaria—IRCCS Istituto Tumori Giovanni Paolo II, Via Orazio Flacco 65, 70124 Bari, Italy; r.massafra@oncologico.bari.it (R.M.); n.petruzzellis@oncologico.bari.it (N.P.); 6 Obstetrics and Gynecology Unit, Department of Biomedical Sciences and Human Oncology, University of Bari Aldo Moro, Piazza Giulio Cesare 11, 70124 Bari, Italy; francescaarezzo@libero.it; 7 Dipartimento di Diagnostica per Immagini, Radioterapia Oncologica ed Ematologia, Fondazione Policlinico Universitario A. Gemelli IRCCS, 00168 Roma, Italy; luca.boldrini@policlinicogemelli.it; 8 Struttura Semplice Dipartimentale di Radiodiagnostica Senologica—IRCCS Istituto Tumori Giovanni Paolo II, Via Orazio Flacco 65, 70124 Bari, Italy; d.laforgia@oncologico.bari.it

**Keywords:** Contrast-Enhanced Mammography (CEM), Dynamic Contrast Magnetic Resonance Imaging (DCE-MRI), radiomics, artificial intelligence

## Abstract

**Simple Summary:**

The assessment of breast lesions through mammographic images is currently challenging, especially in dense breasts. Contrast-enhanced mammography has been shown to overcome the limitations of standard mammography but it greatly depends on the interpretative skills of the physician. The aim of this study was to evaluate the potentialities of statistical and artificial intelligence algorithms as a tool for helping the radiologists in the interpretation of images. The most remarkable results were achieved in discriminating benign from malignant lesions and in the identification of the presence of the hormone receptor. A tool to support the physician’s decision-making process may be designed starting from simple logistic regression and tree-based algorithms. This type of tool may help the radiologist in assessing the investigated breast and in choosing the appropriate follow-up without resorting to histology.

**Abstract:**

Purpose: To evaluate radiomics features in order to: differentiate malignant versus benign lesions; predict low versus moderate and high grading; identify positive or negative hormone receptors; and discriminate positive versus negative human epidermal growth factor receptor 2 related to breast cancer. Methods: A total of 182 patients with known breast lesions and that underwent Contrast-Enhanced Mammography were enrolled in this retrospective study. The reference standard was pathology (118 malignant lesions and 64 benign lesions). A total of 837 textural metrics were extracted by manually segmenting the region of interest from both craniocaudally (CC) and mediolateral oblique (MLO) views. Non-parametric Wilcoxon–Mann–Whitney test, receiver operating characteristic, logistic regression and tree-based machine learning algorithms were used. The Adaptive Synthetic Sampling balancing approach was used and a feature selection process was implemented. Results: In univariate analysis, the classification of malignant versus benign lesions achieved the best performance when considering the original_gldm_DependenceNonUniformity feature extracted on CC view (accuracy of 88.98%). An accuracy of 83.65% was reached in the classification of grading, whereas a slightly lower value of accuracy (81.65%) was found in the classification of the presence of the hormone receptor; the features extracted were the original_glrlm_RunEntropy and the original_gldm_DependenceNonUniformity, respectively. The results of multivariate analysis achieved the best performances when using two or more features as predictors for classifying malignant versus benign lesions from CC view images (max test accuracy of 95.83% with a non-regularized logistic regression). Considering the features extracted from MLO view images, the best test accuracy (91.67%) was obtained when predicting the grading using a classification-tree algorithm. Combinations of only two features, extracted from both CC and MLO views, always showed test accuracy values greater than or equal to 90.00%, with the only exception being the prediction of the human epidermal growth factor receptor 2, where the best performance (test accuracy of 89.29%) was obtained with the random forest algorithm. Conclusions: The results confirm that the identification of malignant breast lesions and the differentiation of histological outcomes and some molecular subtypes of tumors (mainly positive hormone receptor tumors) can be obtained with satisfactory accuracy through both univariate and multivariate analysis of textural features extracted from Contrast-Enhanced Mammography images.

## 1. Introduction

Mammography is one of the main techniques in the diagnosis of breast cancer, showing a key role in both screening and follow-up [1,2]. Mammographic screening has been shown to be highly accurate in detection of breast lesions; however, it suffers from some limitations, especially in the case of dense breasts. In fact, dense breasts show a hyper-intense signal over the mammary parenchyma, resulting in very little contrast between the latter and the lesions. For the mammographic screening of patients with dense breasts, other techniques, such as Magnetic Resonance Imaging (MRI), are commonly preferred [3,4]. In particular, one of the most recent novel approaches is Contrast-Enhanced Mammography (CEM). Combining the potential and benefits of Full-Field Digital Mammography (FFDM), CEM has been shown to be highly effective for the detection and the correct staging of cancer, particularly in dense breasts [4,5,6,7,8,9,10]. More specifically, CEM combines the enhancing properties of the intravenous administration of an iodinated contrast medium with the high precision of digital imaging from FFDM; therefore, the neo-vascularity associated with actively growing malignancy is remarkably emphasized. Due to this property, CEM is not only able to detect cancer with high accuracy, but it is also a powerful technique for the identification of cancers that are obscure at mammography; furthermore, it allows a more accurate evaluation of the disease extent and offers guidance in the planning of surgery and treatment [4,5,6,7,8,9,10]. However, as in all imaging techniques, the evaluation of CEM images depends on the experience and skills of the radiologist, making the identification of automated or semi-automated techniques, which can provide decision support, a considerable challenge.

Recent significant advancements in this sense rely on the application of artificial intelligence and radiomics for the processing of large quantities of data by different imaging modalities [11,12].

Radiomics is the process of extracting quantitative properties, named features, from medical images. This feature extraction generally includes pattern recognition algorithms and provides, as a result, a set of numbers, each representing a quantitative description of a specific either geometrical or physical property of the image portion under consideration. In the context of tumor characterization, the radiomics features typically considered are those that describe properties related to size, shape, intensity, and texture of the tumor [13,14,15,16,17,18,19,20,21,22,23,24,25,26,27].

Biological and molecular features related to breast cancer are commonly extracted by biopsy, which is invasive and not always able to detect tumor heterogeneity [28]. In recent years, there has been growing interest in non-invasive methods to directly derive insights from radiologic images. In this context, the radiomics analysis of tumor features extracted from CEM represents an important tool for breast tumor characterization. As several authors suggest, radiomics analysis combined with artificial intelligence techniques can be used to create a tool to support the physician’s decision-making process in the classification of breast cancer [29,30,31,32,33,34,35,36,37,38,39,40,41,42,43,44]. In fact, through an appropriate tool, the physician would be able to discriminate the tumor nature and/or grading, identifying the adequate treatment for a single patient (e.g., neoadjuvant therapy) or even a more conservative approach (e.g., wait-and-see or conservative surgery). However, based on our knowledge, only some recent studies have used CEM in the prediction of histological grading and receptor status of breast cancer [45,46].

This work aimed to evaluate radiomics features to differentiate malignant versus benign lesions, to predict low versus moderate and high grading, to identify positive or negative hormone receptors, and to discriminate positive versus negative human epidermal growth factor receptor 2 related to breast cancer.

## 2. Methods

### 2.1. Patient Selection

From October 2017 to December 2021, according to regulations issued by the local Institutional Review Board, 182 patients (mean age ± standard deviation of 55.3 ± 10.9 years (range 31–80)) with known breast lesions were enrolled retrospectively. All women signed informed consent. 

Inclusion criteria: patients with known breast lesions (from radiological or clinical screening, symptom of palpable lesions), histologically proven, and that underwent dual-energy CEM. CEM images of patients were acquired at Istituto Nazionale Tumori-IRCCS-Fondazione G. Pascale (Naples, Italy) and at Istituto Tumori “Giovanni Paolo II” of Bari (Bari, Italy).

Exclusion criteria: patient with breast implants, presence of non-removable drilling at the nipple, pacemakers, clips or other metal implants, pregnancy or possible pregnancy, inability to keep upright immobility during the examination, renal disease, or chemotherapy treatment at the time of imaging [41].

Overall, 118 malignant lesions and 64 benign lesions were analyzed.

### 2.2. Imaging Protocol

A total of 136 CEM examinations were performed using the Selenia^®^ Dimensions^®^ Unit dual-energy mammography system (Hologic, Bedford, MA, USA), whereas the remaining 46 CEM image were acquired with the Senographe Essential dual-energy mammography system (GE Healthcare, Princeton, NJ, USA).

The same acquisition protocol was implemented for all the images using both scanners. Specifically, two minutes after the intravenous injection of 1.5 mL/(kg bw) of iodinated contrast medium (Visipaque 320; GE Healthcare, Inc., Princeton, NJ, USA) at a rate of 2–3 mL/s, a set of images was acquired in quick succession, in both CC and MLO views. The CEM examination obtained two images: a low-energy (LE) acquisition at 26–30 kVp and a high-energy (HE) acquisition at 45–49 kVp, depending on breast density and thickness. CEM acquisition details were reported in previous studies [41,42,45].

### 2.3. Image Processing

Two expert radiologists, with 25 and 20 years of experience in breast imaging, manually segmented images by drawing slice-by-slice the contours of the lesions where contrast uptake was emphasized both in CC and MLO views. 

#### MRI Post-Processing with PyRadiomics Tool

For each region of interest, 837 radiomics features were extracted as median values using the PyRadiomics Python package [47] including: First Order Statistics, Grey Level Co-occurrence Matrix, Grey Level Run Length Matrix, Grey Level Size Zone Matrix, Neighboring Grey Tone Difference Matrix, and Grey Level Dependence Matrix features before and after the wavelet filtering. The extracted features comply with feature definitions as described by the Imaging Biomarker Standardization Initiative (IBSI) [48] and as reported in (https://readthedocs.org/projects/pyradiomics/downloads/, accessed on 20 January 2017). 

We used wavelet filtering, with all possible combinations of both high-pass (H) and low-pass (L) filters along the three axes (X, Y, and *Z* axes), to derive six different matrices:

- First Order (FIRST ORDER): Describes the individual values of voxels obtained as a result of ROI cropping. These are generally histogram-based properties (energy, entropy, kurtosis, skewness).
- Gray Level Co-occurrence Matrix (GLCM): Calculates how often the same and similar pixel values come together in an image and records statistical measurements according to this matrix. These resulting values numerically characterize the texture of the image.
- Gray Level Run Length Matrix (GLRLM): Defined as the number of homogeneous consecutive pixels with the same gray tone and quantifies the gray-level values.
- Gray Level Size Zone Matrix (GLSZM): Describes voxel counts according to the logic of measuring gray-level regions in an image.
- Neighboring Gray Tone Difference Matrix (NGTDM): Digitization of textures obtained from filtered images and their fractal properties.
- Gray Level Dependence Matrix (GLDM): Number of bound voxels at a fidex distance from the central voxel.

**Figure 1:**
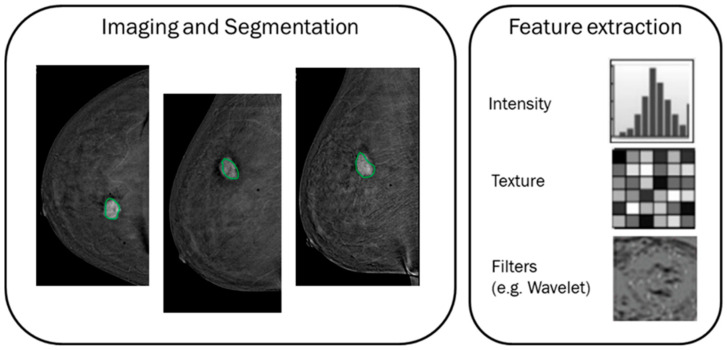
A graphical representation of the features extraction process in a radiomics context. The green lines represent the segmentation of lesion contours.

A graphical representation of the process for features extraction in a radiomics context is reported in Figure 1. 

### 2.4. Histopathological Analysis

The reference standard (ground truth) was the histopathologic examination of tissue as reported in [41]. Breast lesions were categorized based on the American Joint Committee on Cancer staging. The histological grade and the expression of estrogen receptor (ER), progesterone receptor (PR), human epidermal growth factor receptor 2 (HER2), and Ki-67 antigen associated with cell proliferation were determined by immune-histochemical analysis.

The tumor grade G was defined on a three-grade scale according to the Elston–Ellis modification of the Scar–Bloom–Richardson grading system.

The hormone receptor (HR) was also considered; a breast cancer is classified as HR-positive if its cells have receptors for the hormones estrogen and progesterone.

### 2.5. Statistical Analysis

The statistical analysis was performed using the R programming language (version 4.0.2) with the RStudio software, version 1.3.959 (https://www.rstudio.com/, accessed on 20 January 2017) [49].

Considering the histologic results as ground truth, four different types of outcomes were used in both univariate and multivariate analysis: (1) nature of tumor (benign versus malignant); (2) grading (G1 versus G2 + G3); (3) presence of human epidermal growth factor receptor 2 (HER2+ versus HER2−); (4) presence of hormone receptor (HR+ versus HR−).

Before proceeding with statistical analysis, the dataset was balanced with respect of each outcome. The balancing was performed through the synthetization of samples for the less-represented classes using the Adaptive Synthetic Sampling (ADASYN) approach [50,51].

In the context of univariate analysis, the non-parametric Wilcoxon–Mann–Whitney test for continuous variables was used. Receiver operating characteristic (ROC) analysis and the Youden index were considered to obtain the optimal cut-off value for each feature; then, the area under ROC curve (AUC), sensitivity (SENS), specificity (SPEC), positive predictive value (PPV), negative predictive value (NPV), and accuracy (ACC) were computed. Bonferroni correction was used to adjust for multiple comparison.

In the context of multivariate analysis, logistic regression and tree-based algorithms were appropriately designed to predict each outcome individually; the main predictive features were also extracted. Before proceeding with the analysis, three pre-processing steps were performed. 

Firstly, the dataset was randomly split into a training set and a test set, using the createDataPartition R function. Specifically, 90% of the entire dataset was used to train the algorithms, designing a cross-validated procedure; the remaining 10% of samples was used to estimate the accuracy of algorithms on ‘new’ samples, which are samples not used to train the algorithms themselves. Successively (and before running algorithms), a variable selection procedure was designed to remove redundant features from the training set. To achieve this aim, the cross-correlation between each predictor was calculated and all the features with a correlation higher than 0.7 (as an absolute value) with each single predictor were discarded. Finally, the input predictors were centered and scaled before running the logistic regression algorithm.

The machine learning approaches designed for the aim of this paper are described in the following. For each approach, the performance (accuracy) was assessed on both the training and test sets, also considering the values of sensitivity and specificity. 

Logistic Regression. Considering the dichotomic nature of each outcome, a logistic regression was executed using all non-redundant features. The method was run using the glm R function. 

Logistic Regression with least absolute shrinkage and selection operator (LASSO) method. In a different approach, the logistic regression model was fitted on training data, performing a further variable selection with the LASSO regularization method [52,53]. The LASSO was designed using the glmnet R function and the hyperparameter was tuned through a 10-fold cross validation procedure. The variables selected were saved to train the logistic regression algorithm.

Logistic Regression with two predictors. An additional variation of the logistic regression was considered predicting each outcome with all possible couples of features. All combinations that reached a test accuracy higher than 0.9 were saved and analyzed. 

Tree-based algorithms. Among all tree-based algorithms, Classification and Regression Trees (CART) and Random Forest (RF) algorithms were chosen and designed. The CART algorithm was trained taking into account the possibility of obtaining a decision chart, whereas the RF method was used for a more robust evaluation of performances. Tuning of functions’ hyperparameters was performed through a 10-fold cross validation procedure. 

## 3. Results

Table 1 shows the distribution of characteristic of analyzed patients.

Table 2 reports the diagnostic accuracy of significant textural parameters for dual-energy CEM, in both CC and MLO views, obtained in the context of univariate analysis.

As the table shows, in the classification of malignant versus benign lesions, the best performance was reached by the original_gldm_DependenceNonUniformity feature, extracted on CC view, with an accuracy of 89.83%, a sensitivity of 92.37%, and a specificity of 85.59%, and with a cut-off of 2.31.

In the classification of grading, the best performance was reached by the original_glrlm_RunEntropy feature, extracted on CC view, with an accuracy of 83.65%, a sensitivity of 90.38%, and a specificity of 76.92%, and with a cut-off of 0.80.

In the identification of HER2+, the best performance was reached by the wavelet_HLH_gldm_LargeDependenceHighGrayLevelEmphasis feature, extracted on MLO view, with an accuracy of 69.63%, a sensitivity of 62.22%, and a specificity of 77.04%, and with a cut-off of 0.74.

In the identification of HR+, the best performance was reached by the original_gldm_DependenceNonUniformity feature, extracted on CC view, with an accuracy of 81.65%, a sensitivity of 96.99%, and a specificity of 65.59%, and with a cut-off of 2.55.

Table 3 and Table 4 show the results obtained with logistic regression-based and tree-based methods, respectively.

**Figure 2:**
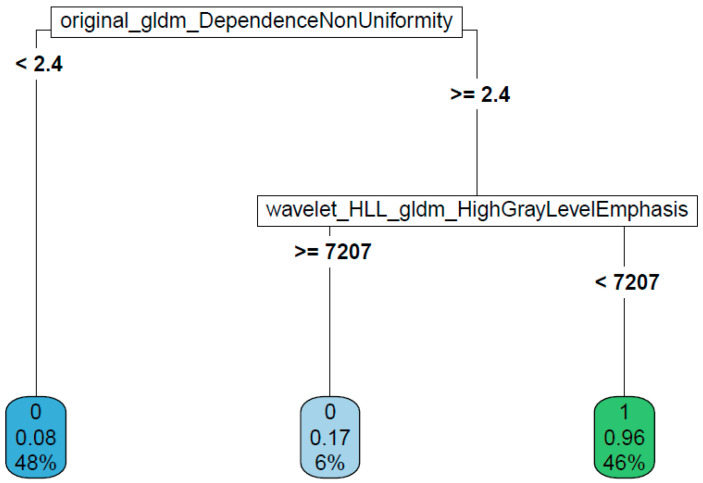
Decisional chart for the prediction of tumor nature from CC images.

**Figure 3:**
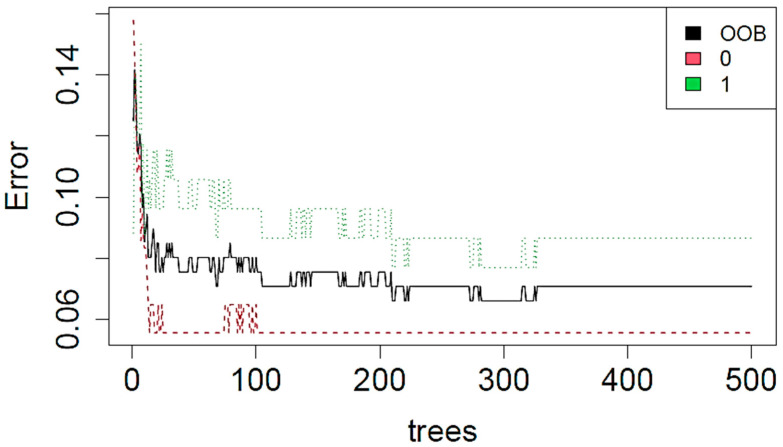
Error evolution during the training procedure of the RF method for the prediction of tumor nature from CC images.

Considering the CC view, the best performances were obtained when predicting the tumor nature (malignant versus benign). Logistic regression proved to be the best performing model (test accuracy of 95.83%) when using an approach without LASSO regularization. The goodness of the logistic regression method was also observed with LASSO regularization (test accuracy of 91.67%). Almost comparable results were obtained when using the tree-based algorithms (Table 5), with a test accuracy of 91.67% in the prediction of tumor nature. The decisional chart obtained with the CART method is shown in Figure 2 and the goodness of training procedure on 500 trees with the RF method is shown in Figure 3.

**Figure 4:**
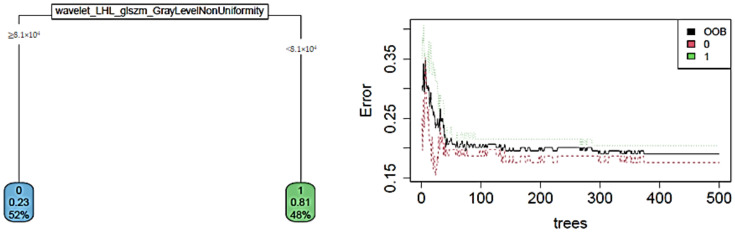
The decision chart and error evolution for the prediction of grading from MLO images.

Considering the features extracted from MLO view images, the best test accuracy (91.67%) was obtained when predicting the grading using a CART algorithm, while the use of all non-redundant features (44 predictors) in a logistic regression model significantly reduces the accuracy value (max test accuracy of 75.00%). The goodness of tree-based algorithms is confirmed by the error evolution plot of RF, reaching an accuracy value of 87.50% on the test set. The decision chart and error evolution are shown in Figure 4.

Combinations of only two features, extracted from both CC and MLO views, always showed test accuracy values greater than or equal to 90.00% (Table 5), with the only exception being the HER2 outcome, where the best performance (test accuracy of 89.29%) was obtained with the RF algorithm.

## 4. Discussions

The radiomics analysis of tumor features extracted from CEM images represents an important tool for breast cancer characterization.

In this study, we aimed to perform radiomics analysis with texture features extracted by dual-energy CEM, evaluating its ability to classify malignant and benign breast lesions, and to predict grading and breast cancer receptors status (HER2+ and HR+).

In recent years, many studies have addressed the problem of breast lesion classification using several feature categories, such as morphological and textural features, in combination with different machine learning approaches, based on CEM and Dynamic Contrast-Enhanced MRI image analysis [29,30,31,32,33,34,35,36,37,38,39,40,54,55,56,57,58], whereas other studies used CEM to predict histological outcomes [45,46].

La Forgia et al. [45] assessed the discrimination power of the statistical features extracted from CEM images to predict histological outcomes and two particular subtypes of tumors, HER2-positive and triple-negative. In their work, they showed encouraging results for the differentiation between ER+/ER−, PR+/PR−, HER2+/HER2−, Ki67+/Ki67−, and High-Grade/Low-Grade. In particular, the highest performances were obtained for discriminating HER2+/HER2− (90.87%), ER+/ER− (83.79%), and Ki67+/Ki67− (84.80%).

In a retrospective study, Marino et al. [46] examined the potential of radiomics analysis using features from both CEM and MRI. In particular, they assessed the tumor invasiveness, the hormone receptor status, and the tumor grade in patients with primary breast cancer through common radiomics parameters. In their results, they showed remarkable accuracies when performing CEM radiomics analysis for discriminating HR+ versus HR− breast cancers (95.6%) and invasive versus non-invasive breast cancers (92.0%); slightly lower results were obtained, instead, in the classification of G1 + G2 versus G3 invasive cancers (77.8%).

The results of the univariate analysis of the present study show that the classification of malignant versus benign lesions achieved the best performance when considering the original_gldm_DependenceNonUniformity feature extracted on CC view (accuracy of 88.98%). The features extracted on CC view appeared to perform better, as the results in the classification of both grading and HR suggest. In fact, an accuracy of 83.65% was reached in the classification of grading, whereas a slightly lower value of accuracy (81.65%) was found in the classification of HR+; the features extracted were the original_glrlm_RunEntropy and the original_gldm_DependenceNonUniformity, respectively. In the identification of HER2+, the best performance, however low (accuracy of 69.63%), was reached when considering the wavelet_HLH_gldm_LargeDependenceHighGrayLevelEmphasis feature, extracted on MLO view images. 

The results of multivariate analysis showed that better performances could be achieved when using two or more features as predictors for the classification of malignant and benign lesions and for the prediction of HR positive status. The best performance was achieved when predicting the tumor nature from the CC images through a logistic regression model, where the test accuracy reached a value of 95.83% without LASSO regulation. Nevertheless, the LASSO regularization selected 12 out of 27 predictors, significantly reducing the model complexity, at the price of an imperceptible reduction in performance (91.67%).

The same performance of logistic regression was not observed when predicting the same outcome (malignant versus benign classification) using MLO images, confirming the tendency of results in the univariate analysis context. 

Features extracted from MLO images were shown to be useful in the prediction of grading with a CART algorithm. However, the results obtained with a logistic regression approach using only two predictors (minimum test accuracy of 90.00%, maximum test accuracy of 95.83%) suggest that simpler models are preferred, with the only exception of the HER2 outcome, where the best performance (test accuracy of 89.29%) was obtained with the RF algorithm.

Remarkable results were also obtained in the prediction of both the grading and the HR+, from both CC and MLO views; however, the performance of logistic regression (regularized or not) and of tree-based algorithms is surpassed by the accuracies obtained when using only two predictive features. Therefore, it can be stated that the prediction of all the outcomes is preferable with less complex models (that is, logistic regression with only two predictors or with a regularized approach). It is furthermore useful to note that the results of univariate analysis are less performant of those of the multivariate approach, suggesting that artificial intelligence can be powerfully used to extract insights from CEM images analysis.

The main limitation of this study is the need for manual segmentation of the images, which is time consuming and operator dependent. The problem of biased results due to this weakness was addressed by having two radiologists perform the segmentation. A foreseeable solution may be the use of automatic or semi-automatic segmentation; however, this may be difficult to implement, especially in the cases of multicentric lesions or background parenchymal enhancement. A further limit is that the interpretation of machine learning algorithm results is not always intuitive and may require specific expertise from the clinician.

## 5. Conclusions

The results of this study confirm that radiomics textural features extracted from CEM images can be highly informative about both the tumor nature and grading, and some molecular subtypes of tumors. Therefore, the results suggest that the combination of artificial intelligence algorithms with the concept of radiomics analysis can be successfully used to create a tool for supporting the physician’s decision-making process in the classification of breast cancer. In particular, the identification of malignant breast lesions and HR positive status can be performed with a high predictive power, even using simpler models.

## Figures and Tables

**Table 1:** Distribution of analyzed patients.

Characteristic	Distribution	
Age	Min value	25
	Max value	82
	Median value	52
Tumor nature	benign	64
	malignant	118
Tumor grading	G1	78
	G2 + G3	104
Human epidermal growth factor receptor 2	HER2+	135
	HER2−	47
Hormone receptor	HR+	93
	HR−	89
Histotype	0	16
	1	2
	2	80
	3	19
	4	14
	5	51

**Table 2:** Performance results of univariate analysis both on CC and MLO view.

Performance Results at Univariate Analysis	Benign Versus Malignant Lesions by CC-View	Benign Versus Malignant Lesions by MLO-View	G1 Versus G2 + G3 by CC-View	G1 Versus G2 + G3 by MLO-View	Identification of HER2+ by CC-View	Identification of HER2+ by MLO-View	Identification of HR+ by CC-View	Identification of HR+ by MLO-View
	original_gldm_DependenceNonUniformity	wavelet_LLL_gldm_DependenceNonUniformity	original_glrlm_RunEntropy	wavelet_LLL_glrlm_RunEntropy	wavelet_HLL_glcm_Idn	wavelet_HLH_glcm_Idm	original_gldm_DependenceNonUniformity	wavelet_LLL_gldm_DependenceNonUniformity
AUC	0.8587	0.8406	0.8237	0.7643	0.7150	0.7081	0.7500	0.7334
SENS	0.9237	0.8220	0.9038	0.7981	0.5481	0.5704	0.9699	0.8495
SPEC	0.8559	0.8814	0.7692	0.7692	0.8148	0.8148	0.6559	0.6882
PPV	0.8651	0.8739	0.7966	0.7757	0.7475	0.7549	0.7355	0.7315
NPV	0.9182	0.8320	0.8889	0.7921	0.6433	0.6548	0.9385	0.8205
ACC	0.8983	0.8517	0.8365	0.7837	0.6815	0.6926	0.8165	0.7688
Cut-off	2.3093	4.1147	0.8023	0.8732	0.8866	0.7384	2.5524	4.2121

**Table 3:** Results for logistic regression with and without LASSO regularization.

Results for Single Outcome	Logistic Regression				Logistic Regression with LASSO			
	Trainset	Test Set			Trainset	Test Set		
	ACC	ACC	SENS	SPEC	ACC	ACC	SENS	SPEC
CC—Tumor nature	0.9583	0.9583	1.0000	0.9286	0.9167	0.9167	0.9000	0.9286
MLO—Tumor nature	0.7500	0.7500	0.8333	0.6667	0.8750	0.8750	1.0000	0.7500
CC—Grading	0.8333	0.8333	0.8571	0.8000	0.7917	0.7917	0.9286	0.6000
MLO—Grading	0.7083	0.7083	0.8462	0.5455	0.7917	0.7917	0.7692	0.8182
CC—HER2	0.7143	0.7143	0.7778	0.6000	0.7857	0.7857	1.0000	0.4000
MLO—HER2	0.6786	0.6786	0.5333	0.8462	0.8214	0.8214	0.8000	0.8462
CC—HR	0.8500	0.8500	0.8182	0.8889	0.8500	0.8500	0.7273	1.0000
MLO—HR	0.7500	0.7500	0.7500	0.7500	0.7000	0.7000	0.5000	1.0000

**Table 4:** Results for CART and RF methods.

Results for Single Outcome	CART				Random Forest			
	Trainset	Test Set			Trainset	Test Set		
	ACC	ACC	SENS	SPEC	ACC	ACC	SENS	SPEC
CC—Tumor nature	0.9122	0.9167	0.9000	0.9286	0.9259	0. 9167	0.9000	0.9286
MLO—Tumor nature	0.8825	0.8333	1.0000	0.6667	0.8968	0.8750	1.0000	0.7500
CC—Grading	0.8073	0.9167	0.9286	0.9000	0.8265	0.8750	0.9286	0.8000
MLO—Grading	0.7660	0.8333	0.8462	0.8182	0.8021	0.8750	0.9231	0.8182
CC—HER2	0.6992	0.6071	0.4444	0.9000	0.7463	0.7143	0.6111	0.9000
MLO—HER2	0.7084	0.8214	0.8667	0.7692	0.8289	0.8929	0.8667	0.9231
CC—HR	0.8045	0.8000	0.6364	1.0000	0.8125	0.8500	0.7273	1.0000
MLO—HR	0.7331	0.7000	0.5000	1.0000	0.7756	0.8000	0.6667	1.0000

**Table 5:** Examples of results for logistic regression methods run using all possible combinations of two predictors.

Results for Single Outcome	ACC	SENS	SPEC	Var 1	Var 2
CC—Tumor nature	0.9583	1.0000	0.9286	original_gldm_SmallDependenceEmphasis	original_firstorder_TotalEnergy
MLO—Tumor nature	0.9167	1.0000	0.8333	original_gldm_LargeDependenceHighGrayLevelEmphasis	wavelet_LHL_glcm_MaximumProbability
CC—Grading	0.9167	0.9286	0.9000	original_gldm_SmallDependenceEmphasis	wavelet_HLL_firstorder_Energy
MLO—Grading	0.9167	1.0000	0.8182	original_glrlm_RunPercentage	original_glszm_LargeAreaLowGrayLevelEmphasis
CC—HR	0.9000	0.8182	1.0000	original_glcm_InverseVariance	original_glcm_DifferenceVariance
MLO—HR	0.9500	0.9167	1.0000	original_firstorder_Maximum	wavelet_LHL_glrlm_RunPercentage

## Data Availability

Data are available at https://zenodo.org/record/6476156#.YmGi2tpBy3A, accessed on 13 March 2022.
